# Effect of High-Carbohydrate Diet on Plasma Metabolome in Mice with Mitochondrial Respiratory Chain Complex III Deficiency

**DOI:** 10.3390/ijms17111824

**Published:** 2016-11-01

**Authors:** Jayasimman Rajendran, Nikica Tomašić, Heike Kotarsky, Eva Hansson, Vidya Velagapudi, Jukka Kallijärvi, Vineta Fellman

**Affiliations:** 1 Folkhälsan Institute of Genetics, Folkhälsan Research Center, 00014 Helsinki, Finland; jayasimman.rajendran@helsinki.fi (J.R.); jukka.kallijarvi@helsinki.fi (J.K.); 2 Institute of Clinical Medicine, Faculty of Medicine, University of Helsinki, 00014 Helsinki, Finland; 3 Department of Clinical Sciences, Lund, Pediatrics, Lund University, 22185 Lund, Sweden; nikica.tomasic@med.lu.se (N.T.); heike.kotarsky@gmail.com (H.K.); eva.hansson@med.lu.se (E.H.); 4 Department of Neonatology, Karolinska University Hospital, 17176 Solna, Sweden; 5 Finnish Institute of Molecular Medicine, University of Helsinki, 00290 Helsinki, Finland; vidya.velagapudi@helsinki.fi; 6 Institute of Clinical medicine, Children’s Hospital, Helsinki University Hospital and University of Helsinki, 00029 Helsinki, Finland

**Keywords:** mitochondrial disorder, *BCS1L*, mouse model, metabolite, dextrose diet, nutrition, GRACILE syndrome

## Abstract

Mitochondrial disorders cause energy failure and metabolic derangements. Metabolome profiling in patients and animal models may identify affected metabolic pathways and reveal new biomarkers of disease progression. Using liver metabolomics we have shown a starvation-like condition in a knock-in (*Bcs1l^c.232A>G^*) mouse model of GRACILE syndrome, a neonatal lethal respiratory chain complex III dysfunction with hepatopathy. Here, we hypothesized that a high-carbohydrate diet (HCD, 60% dextrose) will alleviate the hypoglycemia and promote survival of the sick mice. However, when fed HCD the homozygotes had shorter survival (mean ± SD, 29 ± 2.5 days, *n* = 21) than those on standard diet (33 ± 3.8 days, *n* = 30), and no improvement in hypoglycemia or liver glycogen depletion. We investigated the plasma metabolome of the HCD- and control diet-fed mice and found that several amino acids and urea cycle intermediates were increased, and arginine, carnitines, succinate, and purine catabolites decreased in the homozygotes. Despite reduced survival the increase in aromatic amino acids, an indicator of liver mitochondrial dysfunction, was normalized on HCD. Quantitative enrichment analysis revealed that glycine, serine and threonine metabolism, phenylalanine and tyrosine metabolism, and urea cycle were also partly normalized on HCD. This dietary intervention revealed an unexpected adverse effect of high-glucose diet in complex III deficiency, and suggests that plasma metabolomics is a valuable tool in evaluation of therapies in mitochondrial disorders.

## 1. Introduction

Mitochondrial disorders are caused by mutations or deletions in mitochondrial (mtDNA) or nuclear (nDNA) DNA affecting the respiratory chain or other mitochondrial functions. Mitochondrial dysfunction affects tissues with high energy demand in a highly variable manner at different ages, but neonates are especially vulnerable to low energy availability. Several neonatal mitochondrial disorders present with fetal growth restriction associated with hepatopathy and energy failure, and recently many causative mutations have been identified in specific genes such as *BCS1L*, *DGUOK*, *MPV17*, *POLG*, *SCO1*, and *TFAM* [1,2,3]. *BCS1L* mutations are the most common cause for CIII dysfunction [4]. The most severe *BCS1L*-related disorder is GRACILE syndrome that manifests as fetal onset growth restriction, aminoaciduria due to proximal tubulopathy, hepatopathy with cholestasis and iron overload, lactic acidosis, and early death [5,6,7]. The causative homozygous missense mutation (c.232A>G) resulting in substitution of serine with glycine (p.S78G) in the protein is almost exclusively found in individuals with Finnish ancestry. BCS1L is an assembly factor for respiratory chain (RC) complex III (CIII), needed for the incorporation of the electron-transferring subunit Rieske iron-sulfur protein (RISP) into the complex.

Mitochondrial disorders still lack efficient treatments, and animal models have proven instrumental in investigations of possible therapeutic interventions. We have generated a knock-in mouse model (harboring the *Bcs1l^c.232A>G^* mutation) for GRACILE syndrome. The homozygous mutant mice (*Bcs1l*^G/G^) have early liver glycogen depletion, failure to grow, low blood glucose, and rapid deterioration at about 1 month of age. Liver tissue metabolomics analyses during disease progression showed an initial decrease in carbohydrate intermediates indicating increased glycolysis (Warburg effect) to compensate for decreased ATP production [8].

Recent studies have suggested potential beneficial effects of dietary modification in mitochondrial disorders [9,10,11,12,13,14]. We hypothesized that the fatigue and acute mortality in *Bcs1l*^G/G^ mice is related to hypoglycemia and that by increasing dietary glucose availability their glycemic balance and energy production through glycolysis would be improved. Therefore, we set out to investigate, whether a high carbohydrate diet (HCD) containing dextrose would ameliorate disease severity and improve survival in comparison with normal standard chow diet.

## 2. Results

### 2.1. Reduced Survival and No Effect on Blood Glucose or Weight by High Carbohydrate Diet (HCD) Feeding

**Figure 1:**
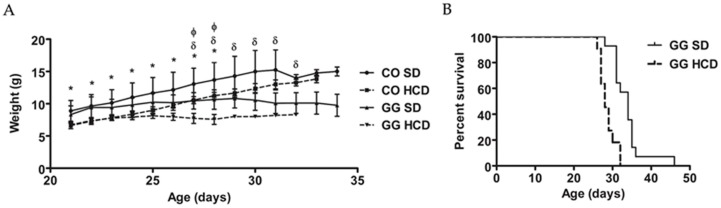
Effect of HCD-feeding (SD, standard diet; HCD, high carbohydrate diet) on weight and survival of *Bcs1l*^G/G^ mice. (**A**) Weight curves of homozygous (GG) and control (CO) mice on respective diets from weaning to deterioration stage (*n* = 4–8/group). Significant differences (*p* < 0.05) are shown for comparing CO on SD with CO on HCD (*) and GG on SD (δ); and CO on HCD with GG on HCD (Φ), Tukey’s multiple comparison; (**B**) survival curve of homozygous mice (GG) on SD (*n* = 30) and HCD (*n* = 21). Median survival was 33 days on SD, 29 days on HCD (*p* < 0.0001, Log-rank, Mental-Cox test).

Entire litters were randomized to receive either HCD or standard diet (SD), and for each homozygous (*Bcs1l*^G/G^) mouse a littermate gender-matched wild-type (WT) or heterozygous (*Bcs1l*^A/G^) mouse was chosen as a control animal (*n* = 51, of which 26 males). Heterozygous mice were included in the control group as their phenotype is identical with that of WT mice. Homozygous mice with matched controls were randomly selected for different assays including blood chemistry and weight measurements (*n* = 6–12 in each group). The homozygous mice had low blood glucose and high lactate-to-glucose ratio, which was unchanged by the increased dietary dextrose (Table 1). HCD had no significant effect on blood ketone levels in control or homozygous (GG) mice as compared to SD. The homozygous mice had reduced weight gain from weaning (at the age of 21 days, P21) to deterioration on SD, HCD unchanged their weight (Figure 1A). Survival (Figure 1B) was significantly shorter on HCD (28 ± 0.6 days, *p* < 0.0001).

### 2.2. No Effect on Hepatic Glycogen Depletion or Fat Accumulation by HCD Feeding

**Figure 2:**
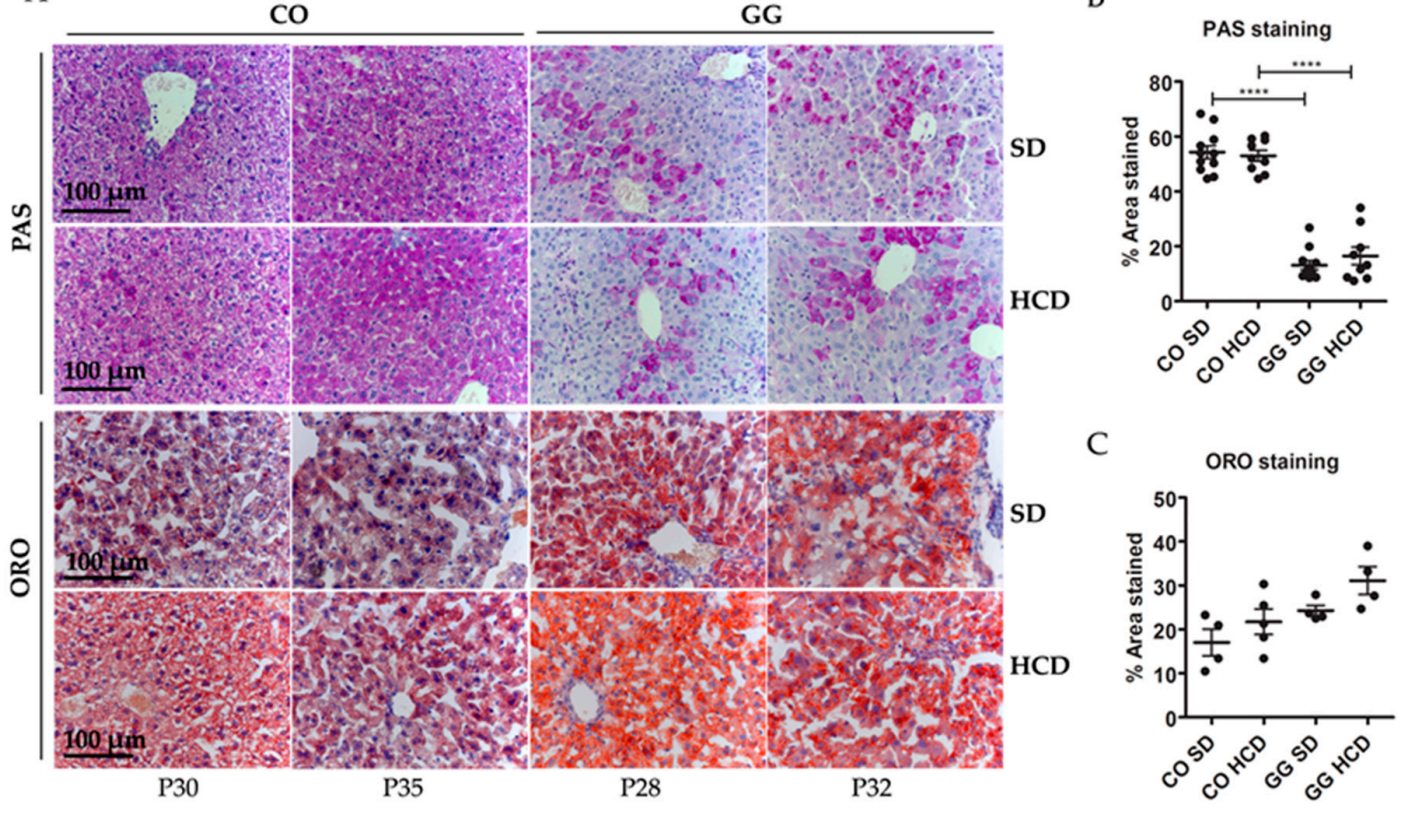
Liver histology. (**A**) Periodic acid–Schiff (PAS) staining showing reduced glycogen in the livers of *Bcs1l^G^*^/*G*^ mutant mice (GG) on both high-carbohydrate (HCD) and standard (SD) diets. Oil Red O (ORO) staining showing slightly increased lipid accumulation in the GG mice on SD and on HCD but the difference was not statistically significant; (**B**,**C**) Image quantification of PAS (*n* = 9–11) and ORO (*n* = 4) staining in the liver sections. *p*-Values: **** *p* < 0.0001.

Histological staining of liver sections revealed hepatopathy at end-stage disease (i.e., deterioration requiring sacrificing) of the homozygous mice. PAS staining showed non-fasting glycogen depletion in homozygotes and this was not affected by HCD. Oil Red O (ORO) staining showed mildly increased lipids in the hepatocytes of the homozygotes and this was not significantly affected by HCD (Figure 2).

### 2.3. No Effect on Respiratory Chain (RC) Complex Assembly or Respiration in Liver Mitochondria by HCD Feeding

**Figure 3:**
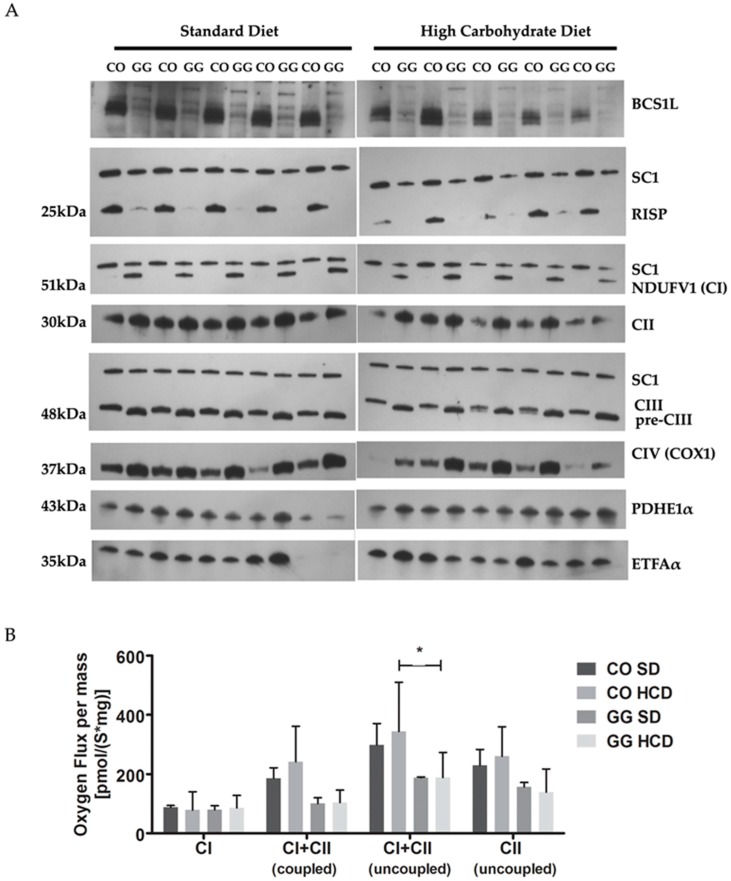
Respiratory chain (RC) complex assembly and respiration in liver mitochondria. (**A**) BNGE analyses show respiratory chain complexes in liver mitochondria as follows: NDUFV1 (CI), NADH dehydrogenase (ubiquinone) complex I; CII, Complex II; CIII, Complex III; CIV, Complex IV; PDHE1α, pyruvate dehydrogenase E1; and ETFAα, electron transport flavoprotein alpha-polypeptide. HCD had no effect on RC complex assembly; (**B**) Liver mitochondrial respiration at coupled and uncoupled state; uncoupling between rate of respiration and ATP production was induced by carbonilcyanide *p*-*tri*-flouro-methoxy-phenyl-hydrazone (FCCP) (*n* = 3–4). HCD had no effect on respiration. SC1, supercomplex. *p*-Values: * *p* < 0.05.

The homozygous *Bcs1l*^c.232A>G^ mutation results in decreased amount of BCS1L and RISP proteins in liver mitochondria [15]. Using Blue Native PAGE (BNGE) and immunoblotting we found no effect of HCD on this depletion. The RISP subunit was localized in supercomplex (SC1) indicating that fully assembled CIII participates in supercomplex formation. The antibody against subunit Core1 detected, in addition, a precomplex of III (pre-CIII) with a smaller molecular size than the fully assembled CIII in control (CO) animals. The homozygous mice had increased amount of complex I (CI) and complex IV (CIV) on SD (Figure 3A). They had low mitochondrial respiration at both coupled (54% of CO) and uncoupled (63% of CO on SD) state and this was not affected by the HCD (Figure 3B).

### 2.4. Altered Plasma Metabolome in Bcs1l Mutant Mice and Partial Normalization of Amino Acid Metabolism and Urea Cycle by HCD Feeding

**Figure 4:**
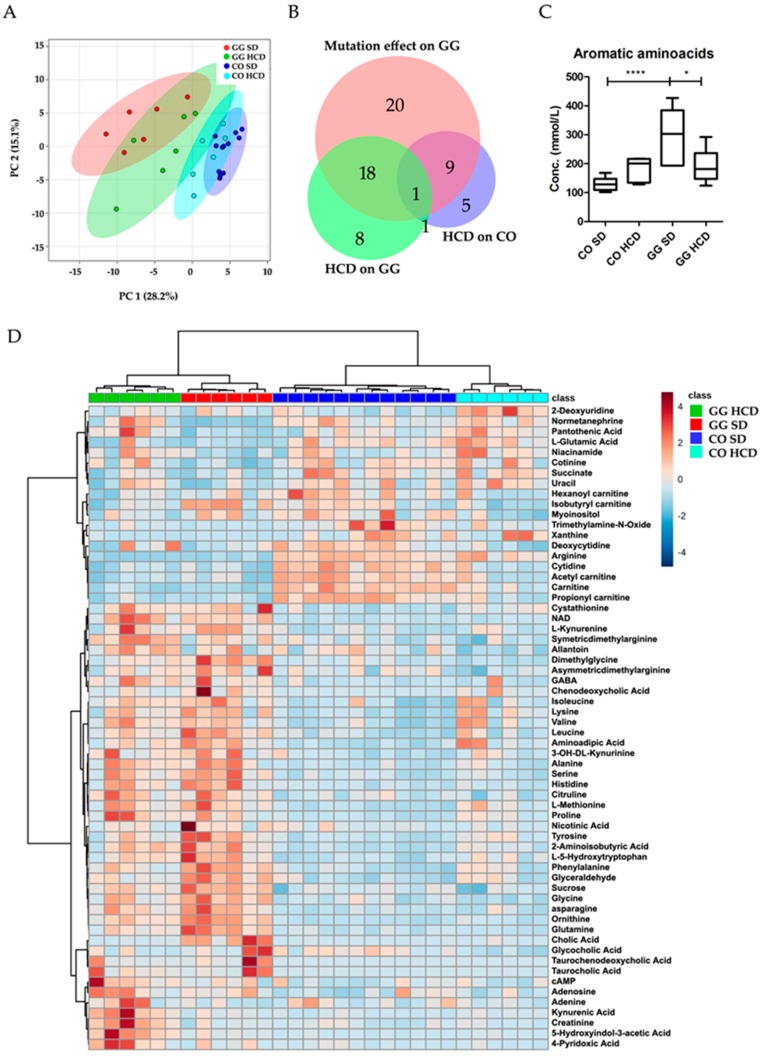
Targeted plasma metabolomics analysis of *Bcs1l*^G/G^ mice on standard and high carbohydrate diet. (**A**) A principle component analysis (PCA) plot showing separation of the feeding groups; (**B**) Area-proportional Venn diagram shows number of metabolites changed in mice due to the mutation and diet, overlap of circles indicates 28 metabolites that were altered by the diet in homozygotes. The diagram was generated using online BioVenn tool; (**C**) Aromatic amino acids (AAA) were normalized by HCD to CO level; (**D**) A heat map of the significantly different metabolites identified from all four mice groups (*n* = 6–7) i.e., control (CO) and homozygous (GG) mice fed with SD and HCD. The graphs show mean ± SD; one-way ANOVA (Tukey’s multiple comparison test); *p*-Values: * *p* < 0.05; **** *p* < 0.0001.

**Figure 5:**
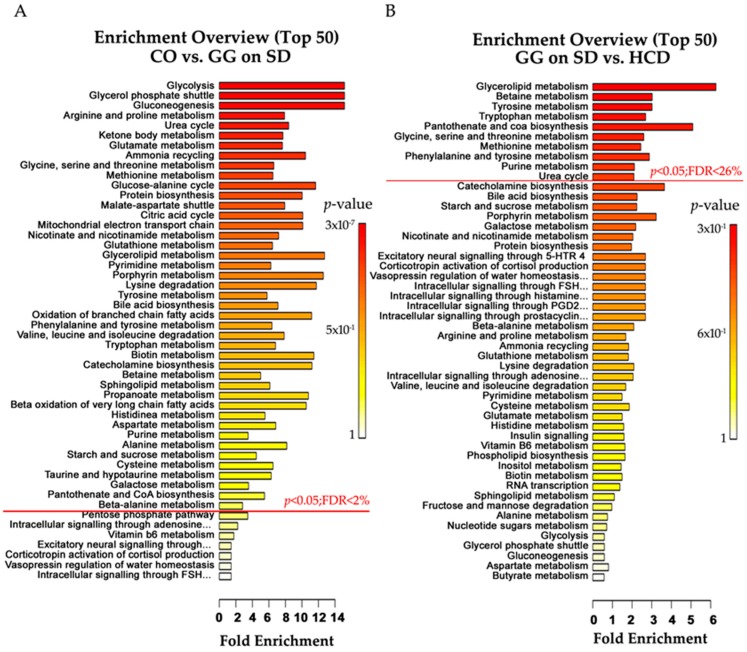
Quantitative enrichment analysis. (**A**) List of pathways in which metabolites were changed by the *Bsc1l^c.232A>G^* mutation in homozygotes on SD; and (**B**) pathways altered by the HCD in homozygotes. Pathways with *p*-value < 0.05 are above the red line and their range of false discovery rate (FDR) is indicated.

Using false discovery rate (FDR) < 10% (*p* < 0.05) in targeted plasma metabolomics analysis, significant alterations were observed in 62/101 metabolites (Table S1) due to the mutation and HCD. The effects on metabolites in the different mouse groups are presented in a Venn diagram (Figure 4B). The mutation alone changed 48 plasma metabolites including amino acid derivatives (23), bile acids (4), neurotransmitter intermediates (3), nucleosides and bases (5), acyl-carnitines (4), urea cycle (2) and other metabolites (7). Most of these were significantly increased in homozygotes on SD, as compared with controls, but some metabolites such as arginine, l-glutamic acid, free and short acyl-carnitines (except isobutyryl-carnitine), normetanephrine, some nucleosides and bases, trimethylamine-*N*-oxide, cotinine and succinate (Figure 4D, Table S1) were decreased. On both diets, homozygous mice had significantly increased sucrose (*p* < 0.001) in plasma (Table 2). Feeding HCD to the homozygotes changed 28 metabolites including 11 amino acids, 3 neurotransmitter intermediates and 14 other metabolites. Kynurenic acid, creatinine, 5-hydroxy-3-indoleacetic acid, 4-pyridoxic acid, and symmetric-dimethyl arginine were higher in homozygotes on HCD than on SD (Table 2). The combined concentration of aromatic amino acids (AAA) was normalized to CO level by HCD in homozygotes (Figure 4C). Quantitative enrichment analysis shows the metabolic pathways that were significantly altered by the mutation (Figure 5A, Table S2) and diet (Figure 5B, Table S3) in *Bcs1l*^G/G^ mice.

## 3. Discussion

The phenotype of the homozygous *Bcs1l^c.232A>G^* mutant mice mimics the clinical characteristics of the GRACILE syndrome, including growth failure, progressive liver disorder with microvesicular steatosis, proximal tubulopathy, and reduced survival [14,16]. Their liver mitochondria show changes in composition of RC complexes: a decreased amount of the BCS1L protein is accompanied by decreased amount of Rieske protein in mitochondria and increased amount of CI and CIV. Furthermore, a precomplex of CIII participates in supercomplex formation but CIV amount is low in supercomplexes [15]. These findings were verified in the present study.

We performed targeted plasma metabolite profiling and show changes in several key metabolites in the homozygous mice. Due to their hypoglycemic state, the homozygotes initially depend on glycogenolysis for energy production, which depletes liver glycogen. As previously shown by liver tissue metabolomics investigation [17], we found a reduction in plasma short-acyl carnitines (except isobutyryl-carnitine), indicating a defect in fatty acid metabolism. Deficits in carbohydrate and fatty acid metabolism are likely the cause of the starvation-like condition and subsequent increased protein catabolism [18]. The associated increases in glucogenic (alanine, asparagine, glycine, dimethylglycine, histidine, methionine, proline, serine and valine) and ketogenic (leucine, isoleucine, phenylalanine and tyrosine) amino acids in circulation support the conclusion of a starvation-like condition in our previous liver metabolomics investigation [17]. The increased plasma aromatic amino acids indicate abnormal liver function and increased protein catabolism, as in patients with liver cirrhosis [19,20]. Moreover, increased alanine, glycine, proline and tyrosine have been considered to be markers of mitochondrial dysfunction in other disease models [21]. 

In skeletal muscle, glucogenic amino acids can be utilized to synthesize glucose and produce pyruvate. Transamination by excessive nitrogen produced from protein degradation converts pyruvate to alanine. The alanine is transported by the blood stream to the liver, where deamination converts it back to pyruvate. This glucose-alanine transport from muscle to liver tissue is known as Cahill cycle [22]. In the *Bcs1l*^G/G^ mice, plasma branched-chain amino acids (BCAA) were increased, which is contrary to the low BCAA levels found in pyruvate dehydrogenase (PDH) deficiency model [21,23]. This suggests that pyruvate to acetyl-CoA conversion may be undisrupted in the *Bcs1l*^G/G^ mice. In the liver, ketogenic amino acids can be utilized to produce ketone bodies and transported to other organs. Krebs cycle intermediates such as acetyl-CoA, alpha-ketoglutarate, succinyl-CoA, fumarates and oxaloacetate can be synthesized from amino acids by transamination and oxidative deamination [24]. Deamination of amino acids releases ammonia which will be processed in the urea cycle. Thus, increased alanine and urea cycle intermediates (citrulline and ornithine) in the homozygous mice suggest activated Cahill cycle and possible hyperammonemia. We found decreased succinate in the plasma, but our previous study showed elevated succinate and fumarate in liver tissue [17], suggesting that circulating succinate is not indicative of its accumulation in the liver.

Unlike liver and muscle, brain tissue can utilize both glucose and ketones as energy source. In order to maintain constant energy supply to the brain, glycogenolysis and gluconeogenesis are carried out in renal proximal tubules as well as in the liver. However, a combined defect in both liver and renal tissue affects glucose homeostasis [8] and thereby energy supply to the brain. During hypoglycemia, brain tissue absorbs tryptophan and produces of 5-hydroxyindoleacetic acid (5-HIAA) and glutamine [16]. We found metabolite changes in the homozygous mice indicating such a derangement, i.e., increased plasma glutamine and 5-hydroxytryptophan (5HTP), an intermediate of 5-HIAA synthesis. Furthermore, we found increased kynurenine (KYN) and nicotinamide adenine dinucleotide (NAD) in homozygous mice. Increased KYN and its derivatives play a role in depression [25], age-associated chronic inflammation [26] and reduced insulin activity [27]. Especially in patients with diabetic retinopathy and other mitochondrial dysfunction, increased levels of KYN and 3-hydroxy kynurenine were found in serum samples [28]. Taken together, our findings suggest that the hypoglycemic condition in the *Bcs1l^G/G^* mutant mice induces a stress resulting in an enhanced tryptophan pathway and increased neurotransmitters such as GABA and 5-HTP. In addition, some of the elevated metabolites have previously been connected to chronic kidney disease, such as significant increases in phenylalanine, N2-succinyl-l-ornithine, l-proline, creatinine and KYN in chronic renal failure [29] and Kidney-Yang deficiency syndrome [30]; increased symmetric dimethylarginine in diabetic nephropathy [31], and asymmetric dimethylarginine accumulation in cardiovascular disease caused by renal failure [32]. Increased kynurenic acid (KYNA) is found in late stage of chronic kidney disease [33]. Increased creatinine is the most common clinical biomarker of renal dysfunction [29,34], also found in Toni–Debré–Fanconi syndrome, which is a typical tubulopathy linked to several different mutations in *BCS1L* in newborn infants [35] and other mitochondrial diseases [36,37]. The increase of these biomarkers in homozygous mice on HCD suggests a more severe renal dysfunction than in mice fed with SD. Interestingly, we found increased sucrose concentrations in the plasma of the homozygous mice. As sucrose is neither synthesized in the body nor absorbed in the intestine, it has been suggested as a marker of gastric mucosal damage leading to non-specific absorption in several animal species [38]. This finding may warrant further investigation of gut mucosal integrity in the *Bcs1l* mutant mice.

In this dietary intervention, the substitution of glucose (dextrose) for corn starch and increasing the total amount of carbohydrate from 44.2% to 65.8% did not improve glycemic balance, growth, or end-stage disease in the mutant mice, but instead caused an unexpected decrease in survival. We chose a HCD with dextrose/glucose instead of sucrose to avoid fructose that may be harmful to the liver at high intake level in mice [39]. High glucose diet is known to induce fatty acid accumulation in liver of wild-type C57BL/6J mice [40] and in carnitine palmityoltransferase 1a deficiency models [10]. Both control and homozygous mice on HCD had marginally increased lipid accumulation in liver as assessed by ORO staining. The liver disease in *Bcs1l* mutant mice is not severe enough to be the cause of death. Thus, a hepatic adverse effect caused by the HCD is unlikely to explain the reduced survival. Protein content was similar in the diets but the HCD contained 24% less (*w*/*w*) fat than the control diet (18.0% vs. 12.9% of energy from fat), which could have adversely affected the energy metabolism in the homozygous mice if there was increased need for β-oxidation due to the RC deficiency. 

HCD did not affect liver glycogen depletion, mitochondrial respiration or CIII assembly, indicating no overall effect on liver disease progression. Given the reduced survival on HCD it was surprising that plasma phenylalanine and tyrosine concentrations were normalized by HCD, suggesting correction of increased protein catabolism. However, HCD feeding resulted in normalization of some amino acids, such as glycine, phenylalanine and tyrosine, which might reduce ammonia release and normalize the urea cycle intermediate ornithine. Our previously liver metabolomics investigation showed signs of oxidative stress at end-stage disease [17]. High-glucose diet has been reported to increase oxidative stress in the liver of wild-type C57BL/6J mice [40]. Here, HCD induced the tryptophan pathway, which can lead to increased synthesis of 5-HIAA and KYNA. KYNA is a metabolite with antioxidant and reactive oxygen species scavenging properties [41]. In addition, significantly increased 4-pyridoxic acid in plasma suggests vitamin B6 catabolism [42], which is normally induced during inflammation to reduce oxidative and aldehyde stress [43]. These changes suggest a response to increased oxidative stress in the *Bcs1l*^G/G^ mice on HCD.

In conclusion, our results suggest that oral dextrose supplementation or high carbohydrate diet formulations may not be efficient in improving the glycemic balance in CIII deficiency and may, in fact, cause adverse effects. Both the *Bcs1l* mutation and the dietary modification had a marked effect on plasma metabolome in the mice, which suggests that targeted plasma metabolomics can be a valuable tool when assessing disease progression and potential therapies in mitochondrial disorders and their animal models.

## 4. Materials and Methods

### 4.1. Animal Experiments

Homozygous mice in congenic C57BL/6 (substrain C57BL/6NCrlLtcf) background were used [15]. Breeding pairs were randomized to HCD using Harlan 60% Dextrose diet (TD05256) or standard Harlan diet (Teklad Globel 18% Rodent Diet—2018) to familiarize the pups from birth with the allocated diet (Table S4). Dextrose is a D-glucose and can be directly absorbed in the intestine. Water was available ad libitum in a vivarium with 12 h light/dark cycle at 22 °C. DNA was isolated from tail biopsies and used for *Bcs1l* genotyping [8]. WT or heterozygous (*Bcs1l^A^*^/*G*^) animals, which do not have any mitochondrial dysfunction, born in the same litter as the homozygotes (*Bcs1l^G^*^/*G*^) were used as controls. A standardized health score was developed based on waddling gait, reduced curiosity, lack of movement in the cage, appearance of kyphosis, deterioration of balance and loss of grip strength. Each behavioral item was scored from zero to two (0 for normal, 1 for slight abnormality and 2 for clear abnormality). When weight gain was decreased compared to the littermate control WT (or heterozygous) mouse, scoring was performed in connection with the daily weighing procedure. When the score was ≥7/12 or no weight gain occurred, the animals were considered as having end-stage disease and were sacrificed before spontaneous death. Mice were anesthetized briefly with isoflurane (792632 Sigma-Aldrich, St. Louis, MO, USA) and blood drops were obtained from the tail artery by pinching with needle for measurement of glucose, lactate and ketone concentrations with Freestyle Lite (Abbot Laboratories, Abbott Park, IL, USA), Lactate Pro (Arkray, Kyoto, Japan) and FreeStyle Precision (Abbot) meters, respectively. The mice were sacrificed by cervical dislocation and thoracic cavity was opened to collect blood with a cardiac puncture. The blood was immediately transferred into Li-heparin vials (Vacuette 454089, Greiner Bio-One International Gmbh, Kremsmunster, Austria). The vials were kept on ice and centrifuged at 2000× *g* for 10 min to isolate plasma, which was stored at −80 °C. 

### 4.2. Ethics Statement

Animal experiments were performed according to national guidelines with the approval of the Lund regional animal research ethics committee (permission M245-11, 19 October 2011). All efforts were taken to ameliorate suffering.

### 4.3. Tissue Sampling

Tissue samples were collected for histology, mitochondrial isolation and snap-freezing, respectively, immediately after sacrificing. The complete data samples were not obtained from each animal due to ethical and practical reasons (See Table S5). Respirometry analyses were performed immediately [15], liver mitochondria isolated, and samples stored at −80 °C for subsequent assays.

### 4.4. Histology

Liver sections were stained with standard methods to assess general histology (hematoxylin-eosin, H & E), glycogen content (PAS, PAS-Diastase), and fat content (Oil Red O, ORO). The area of staining from the image was quantified using Fiji imageJ software (National Institutes of Health, Bethesda, MD, USA) [44].

### 4.5. Respiratory Chain Assessment in Liver Mitochondria by Respirometry and BNGE

From liver specimens mitochondria were isolated by sequential centrifugation including density purification on 19% Percoll (GE Healthcare, Amersham, UK) as previously described [15]. Respiratory chain function was assessed by measuring oxygen consumption in freshly isolated mitochondria using a substrate uncoupler inhibitor titration (SUIT) protocol in Oroboros Oxygraph-2k with DatLab 4 software (Oroboros Instruments, Innsbruck, Austria) as previously described [15]. Structural assessment of mitochondrial complexes and supercomplexes were performed from isolated mitochondria with BNGE as described earlier [15]. Proteins separated by BNGE were blotted onto polyvinylidine difluoride membranes using iBlot™ equipment (Invitrogen, Carlsbad, CA, USA). Membranes were blocked in PBS supplemented with 0.05% Tween 20 and 5% dry milk for subsequent antibody incubation. Most antibodies directed against respiratory chain complex subunits were obtained from MitoSciences (Eugene, OR, USA); for CIII subunits Core1 (MS303) and RISP (MS305) were used, and for CIV subunit I (COX1, MS404). An antibody directed against ETFAα (MS782) was used as loading control. An antibody detecting the CI subunit NDUFV1, which is incorporated in the complex at the final assembly stage, was obtained from Sigma Aldrich (Stockholm, Sweden). Primary antibodies were detected by incubation with HRP-coupled goat anti-mouse secondary antibody (DAKO Cytomation, P0447). Membranes were developed with ECL plus (GE Healthcare, Amersham, UK).

### 4.6. Metabolomics Analysis from Plasma

The isolated blood plasma samples were used to measure 101 targeted metabolites by a triple quadrupole mass spectrometer coupled to ACQUITY UPLC system (Waters Corporation, Milford, MA, USA) in the metabolomics core facility at the Institute for Molecular Medicine Finland (FIMM) [45]. Quantified data were further processed by online tool, MetaboAnalyst 3.0 (McGill University, QC, Canada, http://www.metaboanalyst.ca). Data were auto scaled (mean-centered and divided by the standard deviation of each variable) to perform correlation and enrichment analysis. Unsupervised PCA was used to identify variation in data set. Dendogram was plotted based on Ward and each cell represents concentration values. Bar-graphs were plotted for selected individual metabolites with mean and standard deviation.

### 4.7. Statistics

Statistical differences in blood chemistry, histology, metabolomics were analyzed by one-way ANOVA (Tukeys multiple comparison). Differences in survival curves were analyzed by log-rank, Mental-Cox test. Respirometry data were analyzed by two-way ANOVA (Tukeys multiple comparison). We used GraphPad Prism 5.02 software (GraphPad Software Inc., La Jolla, CA, USA) for analysis and a *p*-value <0.05 was considered significant.

## Figures and Tables

**Table 1:** The effect of a high-carbohydrate diet (HCD) on blood chemistry in control and *Bcs1l^G^*^/*G*^ homozygous mice.

Metabolites (mmol/L)	Control SD (*n* = 38)	Control HCD (*n* = 26)	GG SD (*n* = 30)	GG HCD (*n* = 21)
Glucose	7.0 ± 0.7	6.9 ± 1.2	1.7 ± 0.6 ^1^	2.4 ± 1.6 ^2^
Lactate	4.0 ± 2.2	4.6 ± 1.9	3.5 ± 1.8	4.1 ± 1.2
Lactate/Glucose	0.45 ± 0.2	0.6 ± 0.2	2.0 ± 1.0 ^1^	2.9 ± 2.4 ^2^
Ketones	0.6 ± 0.3	0.8 ± 0.3	0.5 ± 0.2	0.7 ± 0.3

Number of samples varied in each group (*n* = 6 to 12). Data presented as mean ± SD. *p* < 0.05 when compared with (^1^) Control standard diet (SD) and (^2^) Control high carbohydrate diet (HCD); one-way ANOVA and Tukey’s multiple comparison.

**Table 2:** The effect of HCD on plasma metabolomics in control and homozygous (GG) mice.

Metabolites (mmol/L)	Control SD (*n* = 12)	Control HCD (*n* = 6)	GG SD (*n* = 6)	GG HCD (*n* = 6)
**Amino acid derivatives**				
Asymmetricdimethylarginine	4.05 ± 0.7	3.39 ± 0.64	5.55 ± 1.51 ^1^	4.39 ± 0.7
Asparagine	136 ± 29.8	144 ± 75.1	432 ± 164.6 ^1^	311 ± 113 ^2^
Creatinine	5.23 ± 1.2	5.19 ± 0.95	5.6 ± 1.93	13.2 ± 5.4 ^2,3^
Dimethylglycine	8.03 ± 3.7	3.92 ± 1.9	30.1 ± 11.3 ^1^	13.3 ± 4.8 ^3^
Glutamine	918 ± 185	831 ± 65.2	2547 ± 754 ^1^	1457 ± 363 ^2,3^
Glycine	354 ± 116	246 ± 62.8	897 ± 229 ^1^	619 ± 242 ^2,3^
Kynurenic Acid	0.06 ± 0.03	0.102 ± 0.04	0.12 ± 0.08	0.57 ± 0.36 ^2,3^
Leucine	149 ± 50.9	211 ± 117	413 ± 119 ^1^	269 ± 69.9 ^3^
Phenylalanine	43.9 ± 8.8	56.6 ± 13.5	98.0 ± 21.4 ^1^	64.8 ± 16.2 ^3^
Symmetricdimethylarginine	1.3 ± 0.26	1.1 ± 0.42	1.72 ± 0.43	2.3 ± 0.4 ^2,3^
Tyrosine	55.2 ± 11.8	86.5 ± 23.6	164 ± 67.9 ^1^	93.7 ± 37.9 ^3^
**Neurotransmitter intermediates**				
5-Hydroxyindole-3-acetic acid	0.23 ± 0.13	0.32 ± 0.14	0.69 ± 0.47	3.18 ± 1.9 ^2,3^
l-5-Hydroxytryptophan	0.024 ± 0.008	0.04 ± 0.02	0.09 ± 0.04 ^1^	0.06 ± 0.02 ^3^
Normetanephrine	0.018 ± 0.006	0.02 ± 0.006	0.005 ± 0.002 ^1^	0.02 ± 0.012 ^3^
**Others**				
4-pyridoxic acid	0.045 ± 0.03	0.036 ± 0.03	0.11 ± 0.1	0.32 ± 0.23 ^2,3^
Adenine	0.006 ± 0.003	0.005 ± 0.001	0.005 ± 0.001	0.009 ± 0.005
Adenosine	0.05 ± 0.03	0.05 ± 0.02 ^1^	0.08 ± 0.025	0.10 ± 0.05 ^1,2^
cAMP	0.007 ± 0.003	0.007 ± 0.002	0.01 ± 0.003	0.015 ± 0.007 ^1,2^
Chanodeoxycholic acid	41.7 ± 20.4	472 ± 717	1295 ± 1578 ^1^	257 ± 356
Cholic Acid	9.8 ± 11.5	25.1 ± 38.7	428 ± 228.6 ^1^	23.4 ± 14.24 ^3^
Cotinine	0.011 ± 0.003	0.01 ± 0.005	0.005 ± 0.002 ^1^	0.01 ± 0.005 ^3^
Glyceraldehyde	48.9 ± 14.6	67 ± 21.5	115 ± 27.01 ^1^	71.9 ± 12 ^3^
Glycocholic acid	0.46 ± 0.14	0.21 ± 0.07	0.67 ± 0.49 ^2^	0.36 ± 0.16
Isobutyryl carnitine	0.037 ± 0.01	0.022 ± 0.01	0.06 ± 0.02 ^1^	0.024 ± 0.01 ^3^
Nicotinic acid	0.11 ± 0.06	0.08 ± 0.04	0.29 ± 0.29	0.12 ± 0.57
Ornithine	71.8 ± 11.3	86.5 ± 20.7	297 ± 75.4 ^1^	190 ± 43.8 ^3^
Pantothenic acid	3.9 ± 1.14	5.4 ± 1.6	2.56 ± 0.73	5.64 ± 2.4 ^3^
Sucrose	0.2 ± 0.1	0.2 ± 0.1	0.5 ± 0.1 ^1^	0.4 ± 0.1 ^2^

Data are presented as mean ± SD. *p* < 0.05 when compared with Control SD (^1^), Control HCD (^2^) and GG SD (^3^). One-way ANOVA and Tukey’s multiple comparisons.

## Supplementary Materials

Supplementary materials can be found at www.mdpi.com/1422-0067/17/11/1824/s1.
