# Supplementary material for: Effect of High-Carbohydrate Diet on Plasma Metabolome in Mice with Mitochondrial Respiratory Chain Complex III Deficiency
Source: Int J Mol Sci. 2016 Nov 1;17(11):1824. doi: 10.3390/ijms17111824 (PMC5133825; doi:10.3390/ijms17111824)
Supplement: Supplementary file 1 [file ijms-17-01824-s001.zip › ijms-147906-Supplementary Materials/ijms-147906-supple-Tables S1-S4.pdf]

Supplementary Materials: Effect of High-Carbohydrate Diet on Plasma Metabolome in Mice with Mitochondrial Respiratory Chain Complex III Deficiency

Jayasimman Rajendran, Nikica Tomašić, Heike Kotarsky, Eva Hansson, Vidya Velagapudi, Jukka Kallijärvi and Vineta Fellman

Table S1. One-way ANOVA analysis for Plasma metabolomics.

Metabolites	*f* Value	*p* Value	Log *p*	FDR	Fisher's LSD
Ornithine	49.494	6.94×10^{-11}	10.159	7.01×10^{-9}	GG SD-GG HCD; GG SD-CO SD; GG SD-CO HCD; GG HCD-CO SD; GG HCD-CO HCD
Carnitine	44.053	2.47×10^{-10}	9.6079	1.25×10^{-8}	CO SD-GG SD; CO HCD-GG SD; CO SD-GG HCD; CO HCD-GG HCD; CO SD-CO HCD
Arginine	32.735	5.57×10^{-9}	8.2545	1.87×10^{-7}	CO SD-GG SD; CO HCD-GG SD; CO SD-GG HCD; CO HCD-GG HCD
Glutamine	27.743	2.91×10^{-8}	7.5363	7.34×10^{-7}	GG SD-GG HCD; GG SD-CO SD; GG SD-CO HCD; GG HCD-CO SD; GG HCD-CO HCD
CholicAcid	26.017	5.43×10^{-8}	7.2652	1.10×10^{-6}	GG SD-GG HCD; GG SD-CO SD; GG SD-CO HCD
DimethylGlycine	23.703	1.32×10^{-7}	6.8802	2.22×10^{-6}	GG SD-GG HCD; GG SD-CO SD; GG SD-CO HCD; GG HCD-CO HCD
Glycine	19.92	6.48×10^{-7}	6.1882	9.36×10^{-6}	GG SD-GG HCD; GG SD-CO SD; GG SD-CO HCD; GG HCD-CO SD; GG HCD-CO HCD
Propionylcarnitine	19.462	7.97×10^{-7}	6.0985	9.96×10^{-6}	CO SD-GG SD; CO SD-GG HCD; CO SD-CO HCD
Phenylalanine	19.191	9.02×10^{-7}	6.0446	9.96×10^{-6}	GG SD-GG HCD; GG SD-CO SD; GG SD-CO HCD; GG HCD-CO SD
Sucrose	18.998	9.86×10^{-7}	6.006	9.96×10^{-6}	GG SD-GG HCD; GG SD-CO SD; GG SD-CO HCD; GG HCD-CO SD; GG HCD-CO HCD
Cytidine	18.172	1.45×10^{-6}	5.8377	1.33×10^{-5}	CO SD-GG SD; CO SD-GG HCD; CO SD-CO HCD
5-Hydroxyindole-3-aceticacid	17.48	2.03×10^{-6}	5.6926	1.71×10^{-5}	GG HCD-GG SD; GG HCD-CO SD; GG HCD-CO HCD
Glyceraldehyde	17.241	2.28×10^{-6}	5.6416	1.77×10^{-5}	GG SD-GG HCD; GG SD-CO SD; GG SD-CO HCD; GG HCD-CO SD
NAD	16.722	2.95×10^{-6}	5.5295	2.13×10^{-5}	GG SD-CO SD; GG SD-CO HCD; GG HCD-CO SD; GG HCD-CO HCD
Asparagine	15.817	4.70×10^{-6}	5.3283	3.16×10^{-5}	GG SD-GG HCD; GG SD-CO SD; GG SD-CO HCD; GG HCD-CO SD; GG HCD-CO HCD
SymmetriS Dimethylarginine	15.581	5.31×10^{-6}	5.2746	3.35×10^{-5}	GG HCD-GG SD; GG SD-CO SD; GG SD-CO HCD; GG HCD-CO SD; GG HCD-CO HCD
2-Aminoisobutyricacid	14.841	7.89×10^{-6}	5.1031	4.69×10^{-5}	GG SD-CO SD; GG SD-CO HCD; GG HCD-CO SD; GG HCD-CO HCD
Acetylcarnitine	14.725	8.40×10^{-6}	5.0756	4.71×10^{-5}	CO SD-GG SD; CO SD-GG HCD; CO SD-CO HCD
KynurenicAcid	14.029	1.23×10^{-5}	4.9087	6.56×10^{-5}	GG HCD-GG SD; GG HCD-CO SD; GG HCD-CO HCD
Serine	13.916	1.31×10^{-5}	4.8811	6.64×10^{-5}	GG SD-CO SD; GG SD-CO HCD; GG HCD-CO SD; GG HCD-CO HCD

Table S1. *Cont.*

Metabolites	*f* Value	*p* Value	Log *p*	FDR	Fisher's LSD
Creatinine	13.723	1.47×10^{-5}	4.8337	7.05×10^{-5}	GG HCD-GG SD; GG HCD-CO SD; GG HCD-CO HCD
L-5-Hydroxytryptophan	12.998	2.23×10^{-5}	4.6519	9.98×10^{-5}	GG SD-GG HCD; GG SD-CO SD; GG SD-CO HCD; GG HCD-CO SD
Leucine	12.965	2.27×10^{-5}	4.6437	9.98×10^{-5}	GG SD-GG HCD; GG SD-CO SD; GG SD-CO HCD; GG HCD-CO SD
Tyrosine	11.99	4.08×10^{-5}	4.3895	1.72×10^{-4}	GG SD-GG HCD; GG SD-CO SD; GG SD-CO HCD; GG HCD-CO SD
2-deoxyuridine	11.725	4.81×10^{-5}	4.3182	1.94×10^{-4}	CO HCD-GG SD; CO HCD-GG HCD; CO HCD-CO SD
Alanine	10.299	1.2×10^{-4}	3.9199	4.67×10^{-4}	GG SD-CO SD; GG SD-CO HCD; GG HCD-CO SD; GG HCD-CO HCD
Histidine	10.105	1.37×10^{-4}	3.8636	5.12×10^{-4}	GG SD-CO SD; GG SD-CO HCD; GG HCD-CO SD; GG HCD-CO HCD
4-PyridoxicAcid	8.9863	2.97×10^{-4}	3.5273	1.071×10^{-3}	GG HCD-GG SD; GG HCD-CO SD; GG HCD-CO HCD
Citrulline	8.7693	3.47×10^{-4}	3.4597	1.208×10^{-3}	GG SD-CO SD; GG SD-CO HCD; GG HCD-CO SD; GG HCD-CO HCD
Lysine	8.6957	3.66×10^{-4}	3.4366	1.23×10^{-3}	GG SD-CO SD; GG HCD-CO SD; CO HCD-CO SD
Isobutyrylcarnitine	8.6157	3.88×10^{-4}	3.4114	1.23×10^{-3}	GG SD-GG HCD; GG SD-CO SD; GG SD-CO HCD; CO SD-CO HCD
L-Methionine	8.6088	3.9×10^{-4}	3.4092	1.23×10^{-3}	GG SD-CO SD; GG HCD-CO SD; CO HCD-CO SD
Proline	7.8377	6.91×10^{-4}	3.1605	2.115×10^{-3}	GG SD-CO SD; GG HCD-CO SD; GG HCD-CO HCD
AminodipicAcid	7.7256	7.53×10^{-4}	3.1234	2.236×10^{-3}	GG SD-CO SD; GG HCD-CO SD; CO HCD-CO SD
Normetanephrine	7.6403	8.03×10^{-4}	3.0951	2.318×10^{-3}	GG HCD-GG SD; CO SD-GG SD; CO HCD-GG SD
Valine	7.0742	1.249×10^{-3}	2.9034	3.504×10^{-3}	GG SD-CO SD; GG HCD-CO SD; CO HCD-CO SD
Cystathionine	6.8357	1.511×10^{-3}	2.8208	4.124×10^{-3}	GG SD-CO SD; GG SD-CO HCD; GG HCD-CO SD
L-Kynurenine	6.1515	2.648×10^{-3}	2.5771	7.038×10^{-3}	GG SD-CO SD; GG SD-CO HCD; GG HCD-CO SD
Asymmetric-Dimethylarginine	6.1017	2.761×10^{-3}	2.5589	7.15×10^{-3}	GG SD-GG HCD; GG SD-CO SD; GG SD-CO HCD
Allantoin	5.7268	3.796×10^{-3}	2.4207	9.585×10^{-3}	GG SD-CO HCD; GG HCD-CO SD; GG HCD-CO HCD; CO SD-CO HCD
PantothenicAcid	5.5058	4.595×10^{-3}	2.3377	1.132×10^{-2}	GG HCD-GG SD; CO HCD-GG SD; GG HCD-CO SD
cAMP	5.4261	4.926×10^{-3}	2.3075	1.1846×10^{-2}	GG HCD-GG SD; GG HCD-CO SD; GG HCD-CO HCD
Isoleucine	5.3479	5.276×10^{-3}	2.2777	1.2393×10^{-2}	GG SD-CO SD; GG HCD-CO SD
L-GlutamicAcid	5.1635	6.21×10^{-3}	2.2069	1.4256×10^{-2}	CO SD-GG SD; CO HCD-GG SD; CO HCD-GG HCD
GABA	4.9664	7.408×10^{-3}	2.1303	1.6626×10^{-2}	GG SD-CO SD; GG HCD-CO SD; GG HCD-CO HCD
3-Hydroxy-DL-kynurenine	4.9305	7.651×10^{-3}	2.1163	1.68×10^{-2}	GG SD-CO SD; GG SD-CO HCD; GG HCD-CO SD
Adenosine	4.8552	8.19×10^{-3}	2.0867	1.7601×10^{-2}	GG HCD-CO SD; GG HCD-CO HCD
Succinate	4.2698	1.4064×10^{-2}	1.8519	2.9593×10^{-2}	CO SD-GG SD; CO HCD-GG SD; CO SD-GG HCD; CO HCD-GG HCD
deoxycytidine	3.8474	2.1037×10^{-2}	1.677	4.2039×10^{-2}	CO SD-GG SD; CO SD-CO HCD
Taurochenodeoxy-cholicAcid	3.8413	2.1162×10^{-2}	1.6744	4.2039×10^{-2}	GG SD-CO SD; GG SD-CO HCD
GlycocholicAcid	3.8381	2.1227×10^{-2}	1.6731	4.2039×10^{-2}	GG SD-GG HCD; GG SD-CO HCD; CO SD-CO HCD

Table S1. *Cont.*

Metabolites	*f* Value	*p* Value	Log *p*	FDR	Fisher's LSD
Myoinositol	3.7122	2.3984×10^{-2}	1.6201	4.6585×10^{-2}	GG SD-CO HCD; CO SD-CO HCD
Cotinine	3.6899	2.4512×10^{-2}	1.6106	4.6711×10^{-2}	GG HCD-GG SD; CO SD-GG SD; CO HCD-GG SD
TaurocholicAcid	3.6485	2.5525×10^{-2}	1.593	4.7741×10^{-2}	GG SD-CO SD; GG SD-CO HCD; GG HCD-CO SD
Niacinamide	3.6226	2.6179×10^{-2}	1.582	4.8074×10^{-2}	CO HCD-GG SD; CO HCD-CO SD
ChenodeoxycholicAcid	3.5855	0.02715	1.5662	4.8967×10^{-2}	GG SD-GG HCD; GG SD-CO SD
Trimethylamine-*N*-Oxide	3.3463	3.4397×10^{-2}	1.4635	6.095×10^{-2}	CO SD-GG SD; CO SD-GG HCD
Uracil	3.2606	3.7474×10^{-2}	1.4263	6.5257×10^{-2}	CO SD-GG SD; CO HCD-GG SD
Xanthine	3.2286	3.8696×10^{-2}	1.4123	6.6241×10^{-2}	CO SD-GG SD; CO HCD-GG SD; CO SD-GG HCD; CO HCD-GG HCD
Adenine	3.1326	4.2622×10^{-2}	1.3704	7.1747×10^{-2}	GG HCD-GG SD; GG HCD-CO SD; GG HCD-CO HCD
NicotinicAcid	3.1126	4.3492×10^{-2}	1.3616	7.2012×10^{-2}	GG SD-GG HCD; GG SD-CO SD; GG SD-CO HCD
Hexanoylcarnitine	3.0808	4.4914×10^{-2}	1.3476	7.3167×10^{-2}	CO SD-GG HCD; CO SD-CO HCD

FDR—False discovery rate; LSD—Least significant difference; GG—homozygous mice; CO—control mice; SD—standard diet; HCD—High carbohydrate diet; NAD—Nicotinamide adenine dinucleotide; GABA—γ-Aminobutyric acid.

Table S2. Result of quantitative enrichment analysis between Control and homozygous mice on standard diet.

Metabolic Pathways	Total Cmpd	Hits	Statistic q	Expected q	Raw p	Holm p	FDR
Glycolysis	21	1	89.092	5.8824	4.14×10^{-9}	2.57×10^{-7}	8.56×10^{-8}
Glycerol phosphate shuttle	8	1	89.092	5.8824	4.14×10^{-9}	2.57×10^{-7}	8.56×10^{-8}
Gluconeogenesis	27	1	89.092	5.8824	4.14×10^{-9}	2.57×10^{-7}	8.56×10^{-8}
Arginine and proline metabolism	26	8	46.435	5.8824	5.81×10^{-9}	3.43×10^{-7}	9.00×10^{-8}
Urea cycle	20	7	49.351	5.8824	1.07×10^{-8}	6.21×10^{-7}	1.33×10^{-7}
Ketone body metabolism	10	2	45.295	5.8824	2.40×10^{-8}	1.37×10^{-6}	2.48×10^{-7}
Glutamate metabolism	18	7	44.937	5.8824	9.29×10^{-8}	5.20×10^{-6}	8.23×10^{-7}
Ammonia recycling	18	8	61.363	5.8824	1.07×10^{-7}	5.87×10^{-6}	8.27×10^{-7}
Glycine, serine and threonine metabolism	26	9	38.756	5.8824	1.75×10^{-7}	9.44×10^{-6}	1.20×10^{-6}
Methionine metabolism	24	9	38.177	5.8824	4.25×10^{-7}	2.25×10^{-5}	2.64×10^{-6}
Glucose-alanine cycle	12	2	68.551	5.8824	5.42×10^{-7}	2.82×10^{-5}	3.06×10^{-6}
Protein biosynthesis	19	14	58.955	5.8824	8.10×10^{-7}	4.13×10^{-5}	4.05×10^{-6}
Malate-aspartate shuttle	8	3	46.562	5.8824	8.50×10^{-7}	4.25×10^{-5}	4.05×10^{-6}
Citric acid cycle	23	2	59.415	5.8824	1.51×10^{-6}	7.41×10^{-5}	6.25×10^{-6}
Mitochondrial electron transport chain	15	2	59.415	5.8824	1.51×10^{-6}	7.41×10^{-5}	6.25×10^{-6}
Nicotinate and nicotinamide metabolism	13	3	42.225	5.8824	2.65×10^{-6}	1.25×10^{-4}	1.03×10^{-5}
Glutathione metabolism	10	4	37.882	5.8824	2.83×10^{-6}	1.3×10^{-4}	1.03×10^{-5}
Glycerolipid metabolism	13	1	74.897	5.8824	3.51×10^{-6}	1.58×10^{-4}	1.15×10^{-5}
Pyrimidine metabolism	36	6	36.69	5.8824	3.54×10^{-6}	1.58×10^{-4}	1.15×10^{-5}
Porphyrin metabolism	22	1	74	5.8824	4.68×10^{-6}	2.01×10^{-4}	1.45×10^{-5}
Lysine degradation	13	2	69.046	5.8824	5.19×10^{-6}	2.18×10^{-4}	1.53×10^{-5}
Tyrosine metabolism	38	4	33.987	5.8824	6.41×10^{-6}	2.63×10^{-4}	1.81×10^{-5}
Bile acid biosynthesis	49	7	41.706	5.8824	7.81×10^{-6}	3.12×10^{-4}	2.10×10^{-5}
Oxidation of branched chain fatty acids	14	2	65.742	5.8824	1.07×10^{-5}	4.18×10^{-4}	2.77×10^{-5}
Phenylalanine and tyrosine metabolism	13	4	37.466	5.8824	1.13×10^{-5}	4.3×10^{-4}	2.80×10^{-5}
Valine, leucine and isoleucine degradation	36	3	46.168	5.8824	1.41×10^{-5}	5.2×10^{-4}	3.35×10^{-5}
Tryptophan metabolism	34	5	39.984	5.8824	2.88×10^{-5}	1.037×10^{-3}	6.62×10^{-5}
Biotin metabolism	4	1	67.34	5.8824	3.02×10^{-5}	1.057×10^{-3}	6.69×10^{-5}
Catecholamine biosynthesis	5	1	65.975	5.8824	4.23×10^{-5}	1.437×10^{-3}	9.04×10^{-5}
Betaine metabolism	10	5	29.452	5.8824	4.63×10^{-5}	1.527×10^{-3}	9.57×10^{-5}
Sphingolipid metabolism	15	2	35.992	5.8824	7.38×10^{-5}	2.361×10^{-3}	1.48×10^{-4}
Propanoate metabolism	18	1	63.429	5.8824	7.66×10^{-5}	2.373×10^{-3}	1.48×10^{-4}
Beta oxidation of very long chain fatty acids	14	1	61.872	5.8824	1.08×10^{-4}	3.24×10^{-3}	2.03×10^{-4}
Histidine metabolism	11	4	32.538	5.8824	3.86×10^{-4}	1.1186×10^{-2}	7.03×10^{-4}
Aspartate metabolism	12	3	40.198	5.8824	4.05×10^{-4}	1.1344×10^{-2}	7.18×10^{-4}
Purine metabolism	45	12	20.47	5.8824	9.15×10^{-4}	2.4705×10^{-2}	1.576×10^{-3}
Alanine metabolism	6	1	48.009	5.8824	1.434×10^{-3}	3.7285×10^{-2}	2.403×10^{-3}
Starch and sucrose metabolism	14	3	26.402	5.8824	1.503×10^{-3}	3.7572×10^{-2}	2.452×10^{-3}
Cysteine metabolism	8	2	38.202	5.8824	1.611×10^{-3}	3.8652×10^{-2}	2.56×10^{-3}

Table S2. *Cont.*

Metabolic Pathways	Total Cmpd	Hits	Statistic *q*	Expected *q*	Raw *p*	Holm *p*	FDR
Taurine and hypotaurine metabolism	7	1	36.903	5.8824	7.496×10^{-3}	0.1724	1.1618×10^{-2}
Galactose metabolism	25	4	20.806	5.8824	1.3267×10^{-2}	0.29187	2.0062×10^{-2}
Pantothenate and CoA biosynthesis	10	1	32.18	5.8824	1.408×10^{-2}	0.29567	2.0784×10^{-2}
Beta-alanine metabolism	13	4	16.541	5.8824	3.4087×10^{-2}	0.68175	4.9149×10^{-2}
Pentose phosphate pathway	18	1	20.231	5.8824	6.1094×10^{-2}	1	8.6087×10^{-2}
Intracellular signalling through adenosine receptor A2A and adenosine∣intracellular signalling through adenosine receptor A2B and adenosine	7	2	13.071	5.8824	1.1236×10^{-1}	1	1.5481×10^{-1}
Vitamin b6 metabolism	10	2	10.316	5.8824	1.7526×10^{-1}	1	2.3623×10^{-1}
Excitatory neural signalling through through 5-hydroxytryptamine receptor (HTR) 4 and serotonin∣excitatory neural signalling through 5-HTR 7 and serotonin∣excitatory neural signalling through 5-HTR 6 and serotonin	5	1	8.424	5.8824	2.4266×10^{-1}	1	2.8387×10^{-1}
Corticotropin activation of cortisol production	5	1	8.424	5.8824	2.4266×10^{-1}	1	2.8387×10^{-1}
Vasopressin regulation of water homeostasis	5	1	8.424	5.8824	2.4266×10^{-1}	1	2.8387×10^{-1}
Intracellular signalling through FSH receptor and follicle stimulating hormone∣intracellular signalling through LHCGR receptor and luteinizing hormone/ choriogonadotropin	4	1	8.424	5.8824	2.4266×10^{-1}	1	2.8387×10^{-1}
Intracellular signalling through histamine H2 receptor and histamine	5	1	8.424	5.8824	2.4266×10^{-1}	1	2.8387×10^{-1}
Intracellular signalling through PGD2 receptor and prostaglandin D2	5	1	8.424	5.8824	2.4266×10^{-1}	1	2.8387×10^{-1}
Intracellular signalling through prostacyclin receptor and prostacyclin	6	1	8.424	5.8824	2.4266×10^{-1}	1	2.8387×10^{-1}
Folate and pterine biosynthesis	17	2	8.0943	5.8824	2.6581×10^{-1}	1	3.0045×10^{-1}
Fructose and mannose degradation	18	1	7.6504	5.8824	2.6653×10^{-1}	1	3.0045×10^{-1}
Butyrate metabolism	9	1	1.4981	5.8824	6.285×10^{-1}	1	6.8028×10^{-1}
One carbon pool by folate	8	1	1.4576	5.8824	6.3322×10^{-1}	1	6.8028×10^{-1}

Table S2. *Cont.*

Metabolic Pathways	Total Cmpd	Hits	Statistic *q*	Expected *q*	Raw *p*	Holm *p*	FDR
Inositol metabolism	19	2	2.6146	5.8824	6.5431×10^{-1}	1	6.8028×10^{-1}
Insulin signalling	19	3	3.4459	5.8824	6.6305×10^{-1}	1	6.8028×10^{-1}
RNA transcription	9	1	1.1743	5.8824	6.6864×10^{-1}	1	6.8028×10^{-1}
Phospholipid biosynthesis	19	1	1.1693	5.8824	6.693×10^{-1}	1	6.8028×10^{-1}
Nucleotide sugars metabolism	9	1	0.73929	5.8824	7.3444×10^{-1}	1	7.3444×10^{-1}

Table S3. Result of quantitative enrichment analysis between homozygous mice on standard and high carbohydrate diet.

Metabolic Pathways	Total Cmpd	Hits	Statistic *q*	Expected *q*	Raw *p*	Holm *p*	FDR
Glycerolipid metabolism	13	1	56.974	9.0909	0.004545	0.28179	0.17675
Betaine metabolism	10	5	27.483	9.0909	0.008893	0.54247	0.17675
Tyrosine metabolism	38	4	27.407	9.0909	0.009369	0.56214	0.17675
Tryptophan metabolism	34	5	24.445	9.0909	0.012649	0.74632	0.17675
Pantothenate and coa biosynthesis	10	1	46.273	9.0909	0.01492	0.86537	0.17675
Glycine, serine and threonine metabolism	26	9	23.588	9.0909	0.017105	0.97498	0.17675
Methionine metabolism	24	9	22.21	9.0909	0.020219	1	0.17908
Phenylalanine and tyrosine metabolism	13	4	26.169	9.0909	0.024551	1	0.19027
Purine metabolism	45	12	19.265	9.0909	0.04478	1	0.26012
Urea cycle	20	7	19.057	9.0909	0.048986	1	0.26012
Catecholamine biosynthesis	5	1	33.157	9.0909	0.050081	1	0.26012
Bile acid biosynthesis	49	7	20.48	9.0909	0.050346	1	0.26012
Starch and sucrose metabolism	14	3	20.349	9.0909	0.063278	1	0.26596
Porphyrin metabolism	22	1	29.309	9.0909	0.069088	1	0.26596
Galactose metabolism	25	4	19.865	9.0909	0.0857	1	0.26596
Nicotinate and nicotinamide metabolism	13	3	18.557	9.0909	0.10065	1	0.26596
Protein biosynthesis	19	14	17.883	9.0909	0.10079	1	0.26596
Excitatory neural signalling through 5- hydroxytryptamine receptor (HTR) 4 and serotonin∣excitatory neural signalling through 5-HTR 7 and serotonin∣excitatory neural signalling through 5-HTR 6 and serotonin	5	1	24.36	9.0909	0.10295	1	0.26596
Corticotropin activation of cortisol production	5	1	24.36	9.0909	0.10295	1	0.26596
Vasopressin regulation of water homeostasis	5	1	24.36	9.0909	0.10295	1	0.26596
Intracellular signalling through FSH receptor and follicle stimulating hormone∣intracellular signalling through lhcgr receptor and luteinizing hormone/ choriogonadotropin	4	1	24.36	9.0909	0.10295	1	0.26596
Intracellular signalling through histamine H2 receptor and histamine	5	1	24.36	9.0909	0.10295	1	0.26596
Intracellular signalling through PGD2 receptor and prostaglandin D2	5	1	24.36	9.0909	0.10295	1	0.26596
Intracellular signalling through prostacyclin receptor and prostacyclin	6	1	24.36	9.0909	0.10295	1	0.26596

Table S3. *Cont.*

Metabolic Pathways	Total Cmpd	Hits	Statistic *q*	Expected *q*	Raw *p*	Holm *p*	FDR
Beta-alanine metabolism	13	4	18.972	9.0909	0.10911	1	0.27059
Arginine and proline metabolism	26	8	15.267	9.0909	0.11716	1	0.27939
Ammonia recycling	18	8	16.561	9.0909	0.13267	1	0.29522
Glutathione metabolism	10	4	16.468	9.0909	0.13694	1	0.29522
Lysine degradation	13	2	19.026	9.0909	0.13809	1	0.29522
Intracellular signalling through adenosine receptor A2A and adenosine∣intracellular signalling through adenosine receptor A2B and adenosine	7	2	18.655	9.0909	0.14435	1	0.29832
Valine, leucine and isoleucine degradation	36	3	15.254	9.0909	0.16817	1	0.33257
Pyrimidine metabolism	36	6	13.547	9.0909	0.17424	1	0.33257
Cysteine metabolism	8	2	16.869	9.0909	0.17701	1	0.33257
Glutamate metabolism	18	7	13.584	9.0909	0.18558	1	0.33432
Histidine metabolism	11	4	14.319	9.0909	0.19149	1	0.33432
Insulin signalling	19	3	14.45	9.0909	0.19412	1	0.33432
Vitamin B6 metabolism	10	2	14.872	9.0909	0.20242	1	0.33918
Phospholipid biosynthesis	19	1	14.933	9.0909	0.21467	1	0.35025
Inositol metabolism	19	2	13.088	9.0909	0.23776	1	0.37071
Biotin metabolism	4	1	13.545	9.0909	0.23917	1	0.37071
RNA transcription	9	1	12.541	9.0909	0.25873	1	0.39125
Sphingolipid metabolism	15	2	9.9573	9.0909	0.34635	1	0.50254
Fructose and mannose degradation	18	1	8.8205	9.0909	0.34853	1	0.50254
Alanine metabolism	6	1	6.7891	9.0909	0.41339	1	0.5825
Nucleotide sugars metabolism	9	1	6.4467	9.0909	0.42585	1	0.5835
Glycolysis	21	1	5.3214	9.0909	0.47071	1	0.5835
Glycerol phosphate shuttle	8	1	5.3214	9.0909	0.47071	1	0.5835
Gluconeogenesis	27	1	5.3214	9.0909	0.47071	1	0.5835
Aspartate metabolism	12	3	7.2703	9.0909	0.47252	1	0.5835
Butyrate metabolism	9	1	5.2206	9.0909	0.47506	1	0.5835
Glucose-alanine cycle	12	2	6.0553	9.0909	0.47998	1	0.5835
Malate-aspartate shuttle	8	3	5.1521	9.0909	0.54929	1	0.64473
Ketone body metabolism	10	2	5.271	9.0909	0.56027	1	0.64473
One carbon pool by folate	8	1	3.4804	9.0909	0.56154	1	0.64473
Beta oxidation of very long chain fatty acids	14	1	3.0204	9.0909	0.58907	1	0.66404
Citric acid cycle	23	2	3.7354	9.0909	0.63647	1	0.6923
Mitochondrial electron transport chain	15	2	3.7354	9.0909	0.63647	1	0.6923
Folate and pterine biosynthesis	17	2	2.9161	9.0909	0.72298	1	0.77284
Propanoate metabolism	18	1	1.1408	9.0909	0.74111	1	0.77879
Pentose phosphate pathway	18	1	0.5445	9.0909	0.81972	1	0.84704
Oxidation of branched chain fatty acids	14	2	1.5711	9.0909	0.84347	1	0.85729
Taurine and hypotaurine metabolism	7	1	0.075561	9.0909	0.93242	1	0.93242

Table S4. Diet composition of Standard and High carbohydrate diet.

Standard Diet (SD)			High Carbohydrate Diet (HCD)		
**(Teklad Globel 18% Rodent Diet-2018)**			**(60% Dextrose Diet, Orange—TD.05256)**		
Crude Protein	%	18.6	Casein	g/kg	207
Fat	%	6.2	Lard	g/kg	50
Carbohydrate	%	44.2	Dextrose, monohydrate	g/kg	658
Crude Fiber	%	3.5	Cellulose	g/kg	21.81
Neutral Detergent fiber	%	14.7			
Ash	%	5.3			
Energy Density	kcal/g	3.1			
Calories from Protein	%	24	Calories from Protein	% kcal	20.2
Calories from Fat	%	18	Calories from Fat	% kcal	12.9
Calories carbohydrate	%	58	Calories carbohydrate	% kcal	66.8
			Food color	g/kg	0.15
**Minerals**			**Mineral Mix, Rogers-Harper (170760)-50 g/kg**		
Calcium	%	1	Calcium Carbonate	g/kg	292.955
Phosphorus	%	0.7	Calcium Phosphate, dibasic, dihydrate	g/kg	4.3
Non-Phytate Phosphorus	%	0.4	Ammonium Paramolybdate, tetrahydrate	g/kg	0.025
Sodium	%	0.2	Sodium Chloride	g/kg	250.6
Potassium	%	0.6	Potassium Phosphate,monobasic	g/kg	343.1
Chloride	%	0.4	Zinc Chloride	g/kg	0.2
Magnesium	%	0.2	Magnesium Sulfate, heptahydrate	g/kg	99.8
Zinc	mg/kg	70	Zinc Carbonate	g/kg	0.04
Manganese	mg/kg	100	Manganese Sulfate, monohydrate	g/kg	1.21
Copper	mg/kg	15	Cupric Sulfate	g/kg	1.56
Iodine	mg/kg	6	Potassium Iodide	g/kg	0.005
Iron	mg/kg	200	Ferric Citrate	g/kg	6.23
Selenium	mg/kg	0.23	Sodium Selenite	g/kg	0.015
**Vitamins**			**Vitamin Mix, Teklad (40060)-10 g/kg**		
Vitamin A	IU/g	15	Vitamin A Palmitate (500,000 IU/g)	g/kg	3.9648
Vitamin D3	IU/g	1.5	Vitamin D3, cholecalciferol (500,000 IU/g)	g/kg	0.4405
Vitamin E	IU/kg	110	Vitamin E, DL-α tocopheryl acetate (500 IU/g)	g/kg	24.2291
Vitamin K3	mg/kg	50	Vitamin K3, menadione	g/kg	4.9559
Vitamin B1	mg/kg	17	Thiamin (81%)	g/kg	2.2026
Vitamin B2	mg/kg	15	Riboflavin	g/kg	2.2026
Niacin (Nicotinic acid)	mg/kg	70	Niacin	g/kg	9.9119
Vitamin B6	mg/kg	18	Pyridoxine HCl	g/kg	2.2026
Pantothenic Acid	mg/kg	33	Calcium Pantothenate	g/kg	6.6079
Vitamin B12	mg/kg	0.08	Vitamin B12 (0.1% in mannitol)	g/kg	2.9736
Biotin	mg/kg	0.4	Biotin	g/kg	0.0441
Folate	mg/kg	4	Folic Acid	g/kg	0.1982
Choline	mg/kg	1200	Choline Dihydrogen Citrate	g/kg	349.6916
			Vitamin C, (−97.5%)	g/kg	101.6604
			Inositol	g/kg	11.0132
			p-Aminobenzoic Acid	g/kg	11.0132
			Corn Starch	g/kg	466.687

Table S4. *Cont.*

Standard Diet (SD)			High Carbohydrate Diet (HCD)		
**Amino Acids**					
Aspartic Acid	%	1.4			
Glutamic Acid	%	3.4			
Alanine	%	1.1			
Glycine	%	0.8			
Threonine	%	0.7			
Proline	%	1.6			
Serine	%	1.1			
Leucine	%	1.8			
Isoleucine	%	0.8			
Valine	%	90%			
Phenylalanine	%	1			
Tyrosine	%	0.6			
Methionine	%	0.4	DL-Methionine	g/kg	3
Cystine	%	0.3			
Lysine	%	0.9			
Histidine	%	0.4			
Arginine	%	1			
Tryptophan	%	0.2			
**Fatty Acids**					
Palmitic	%	0.7			
Stearic	%	0.2			
Oleic	%	1.2			
Linoleic	%	3.1			
Linolenic	%	0.3			
Total Saturated	%	0.9			
Total Monounsaturated	%	1.3			
Total Polyunsaturated	%	3.4			
